# 多通道非接触电导检测装置用于自由流电泳分离在线检测

**DOI:** 10.3724/SP.J.1123.2021.11011

**Published:** 2022-04-08

**Authors:** Ziqi LIANG, Qiang ZHANG, Xiaoteng JIANG, Xiaoping LIU, Chengxi CAO, Hua XIAO, Weiwen LIU

**Affiliations:** 1.上海交通大学电子信息与电气工程学院, 上海 200240; 1. School of Electronic Information & Electrical Engineering, Shanghai Jiao Tong University, Shanghai 200240, China; 2.上海交通大学生命科学技术学院, 上海 200240; 2. School of Life Sciences and Biotechnology, Shanghai Jiao Tong University, Shanghai 200240, China

**Keywords:** 在线检测, 电容耦合式非接触电导检测, 多通道, 自由流电泳, 蛋白质分离, online detection, capacitively coupled contactless conductivity detection (C^4^D), multi-channel, free-flow electrophoresis (FFE), protein separation

## Abstract

现有自由流电泳(FFE)装置因不具备在线检测功能,其实用性仍然存在明显不足。针对这一问题,该工作发展了一种多通道电容耦合式非接触电导检测(MC-C^4^D)装置并开发了自动测量软件。MC-C^4^D装置采用了并行分时的非接触电导检测技术,即由多个同样的非接触电导检测模块并行排列,而单个电导检测模块又由多个非接触电导检测池组成,采用模拟开关切换这些检测池,能够分时检测流经相应检测池溶液的电导率。多个电导检测模块的检测池总数等于FFE的组分数,它们分别串行接入到FFE各流路中,这样MC-C^4^D装置就可在线并行分时在线测量各组分溶液的电导率。为验证所设计MC-C^4^D装置的检测性能,采用配制的氯化钾标准溶液作为检测对象对MC-C^4^D装置进行了标定和测试。实验数据表明,MC-C^4^D装置电导率检测范围为0.015~2.5 mS/cm,检出限(LOD)为0.002 mS/cm,日内相对标准偏差(RSD, *n*=3)为2.31%,测量相对误差(RE)为3.03%和通道间测量相对偏差为1.60%,这些参数表明该装置检测范围较大,LOD低,重复性好,准确性高,通道间测量相对偏差小。另外,将MC-C ^4^D装置应用于往复式自由流等电聚焦电泳(RFFIEF)在蛋白质聚焦过程中对各组分溶液电导率进行实时在线检测,结果表明,所开发的MC-C^4^D装置不仅可实现对FFE各组分溶液电导率的实时在线检测,而且还可在RFFIEF实验中辅助掌握分离的实验进度,提高FFE装置的实用性。

## Abstract (en)

Free-flow electrophoresis (FFE) is an all-liquid-phase electrophoresis technique without any supporting media, which has both analytical and preparative functions. Compared to other electrophoresis techniques, FFE has been used for the separation of peptides, proteins, cells, and microorganisms due to its advantages of mild separation environment, high recovery, and sustainable separation. Both the online detection of the characteristic parameters for each component solution and the real-time control of the progress of the separation experiment are of considerable importance for the study of FFE separation. Since the existing FFE devices do not have the online detection function, there are obvious deficiencies in their practicability. The absence of online detection function not only made it impossible to track the progress of the separation experiment in real time, but also made it difficult to detect the properties of the component solutions, which still require offline testing after separation. In this study, a multi-channel capacitively coupled contactless conductivity detection (MC-C^4^D) device has been developed to solve this problem, and an automatic measurement software has also been developed. The MC-C^4^D device used a parallel time-sharing contactless conductivity detection technique. It consisted of several contactless conductivity detection modules arranged in parallel, which in turn consisted of a number of contactless conductivity cells that were switched on/off by analog multiplexers for detecting the conductivity of the solution flowing through the cells in real time. The number of cells was equal to the number of components of the FFE. The components were connected to each of the FFE flow channels, such that the MC-C^4^D device could be used to measure the conductivity of the solution flowing through each channel in parallel online. To verify the performance of the MC-C^4^D device, calibration was conducted by using potassium chloride (KCl) standard solutions on MC-C^4^D device. The experimental data showed that the detection range of MC-C^4^D was 0.015-2.5 mS/cm, and the limit of detection (LOD) was 0.002 mS/cm. The intra-day relative standard deviation (RSD, *n*=3) was 2.31%, the measurement relative error (RE) was 3.03%, and the measurement difference between channels was 1.60%. All these data validated that the device had the advantages of wide detection range, low LOD, good repeatability, high accuracy, and low variation between channels. The MC-C ^4^D device was also applied to reciprocating free-flow isoelectric focusing (RFFIEF) electrophoresis for real-time online detection of the conductivity of each component solution during protein focusing. At the start of isoelectric focusing, when the ions had not reached equilibrium loading in the electric field and the pH gradient had not yet been fully developed, there was little difference in conductivity between the different channels and the channel conductivity curve was relatively flat. As the experiment progressed, the proteins gradually started to enrich the anodic end. As the proteins accumulated towards the isoelectric point, their own net charge gradually decreased, and thus, the conductivity of the solution in the channels near the anodic region also decreased. Under sufficient isoelectric focusing, protein enrichment was evident. In the focusing region, the conductivity of the solution in the corresponding channel decreased further. There was also an increase in the conductivity of the solution in the corresponding channel due to the accumulation of ions near the electrode ends. These results showed that the MC-C^4^D device not only enabled real-time online detection of the conductivity of each component solution in FFE, but also aided in mastering the progress of separation experiment in RFFIEF, thus improving the practicality of the FFE device. Thus, the MC-C^4^D device, which had the advantages of good performance, small size, simple circuit system, easy installation and commissioning, and low cost, could play an important role in multi-channel measurement, online inspection, and process monitoring.

自由流电泳(free-flow electrophoresis, FFE)是一种无支持介质的全液相电泳技术,兼具分析和制备两种功能^[1,2]^。与其他电泳技术相比,自由流电泳技术具有分离环境温和、回收率高、可持续分离等优点,已用于多肽、蛋白质、细胞和微生物等的分离^[3,4,5]^。在FFE分离实验中,无论是各组分溶液特性参数的在线检测,还是实时掌握分离的实验进度,对于分离实验的研究都具有非常重要的意义。然而,因管路系统复杂(通道多),现有的FFE装置都不具备在线检测功能,这样不仅无法实时掌握分离的实验进度,而且对各组分溶液特性参数的检测也仍然依赖于分离后再进行离线检测,这种方式极不便利,费工费时,另外在进行细胞的细胞器分离时还尤其不利于细胞器生物活性的维持^[6,7,8,9,10,11]^。

电容耦合非接触式电导检测(capacitively coupled contactless conductivity detection, C^4^D)为解决这些问题开辟了新的道路。1980年,Gaš等^[12]^首先提出了C^4^D的概念,并将其应用于同位素检测。Zemann等^[13,14,15]^在1998年设计了一种轴向双电极的C^4^D装置,用作毛细管电泳分离检测器。在过去的二十年中,由于C^4^D具有灵敏度高、体积小、非接触检测和成本低等优点,其已广泛应用于电泳及液相色谱中对分析物的电导率进行在线检测^[16,17,18,19,20,21,22]^。2001~2002年,Laugere等^[23,24]^提出了四电极的C^4^D装置,通过减少耦合电容的影响,提高了芯片电泳中载体缓冲液电导率的检测范围(0.2~20 mmol/L, 0.014~2.7 mS/cm)。Shih等^[25]^在2006年运用并联谐振的方法,提出了灵敏度提高10000倍的C^4^D检测器,用于检测稀释后低浓度的电解质。然而目前这种单通道C^4^D操作繁琐,无法满足FFE多通道在线检测的要求。如果采用32通道并行检测,则需整体设置32个并行的C^4^D检测器,这样做不但体积非常庞大、电路系统复杂以及安装调试困难,而且也将使得FFE设备的造价极其昂贵。

本工作发展了一种多通道电容耦合式非接触电导检测(multi-channel capacitively coupled contactless conductivity detection, MC-C^4^D)装置。MC-C^4^D装置采用了并行分时的非接触电导检测技术,即由多个同样的非接触电导检测模块并行排列,而单个电导检测模块又由多个非接触电导检测池组成,采用模拟开关切换这些检测池以分时检测流经相应检测池溶液的电导率。多个检测模块的检测池总数等于FFE的组分数,它们分别串行接入到FFE各流路中,这样MC-C^4^D装置就可在线并行分时测量各组分溶液的电导率。为验证所设计装置的检测性能,采用配制的氯化钾(KCl)标准溶液作为检测对象对其进行了标定和测试。另外,将MC-C^4^D装置应用于往复式自由流等电聚焦电泳(reciprocating free-flow isoelectric focusing, RFFIEF)在蛋白质聚焦过程中对各组分溶液电导率进行实时在线检测,结果表明,所开发的MC-C^4^D装置不仅可实时在线检测各组分溶液的电导率,而且还可辅助掌握分离的实验进度,提高了FFE装置的实用性。

## 1 实验部分

### 1.1 仪器

通用电源Pac^TM^ HV(Bio-Rad,美国)用于自由流电泳。带有DJS-1C电导电极的商用接触式电导率仪DDS-307(REX,上海)用于电导检测^[26]^。数据采集卡MP417(北京双诺测控技术有限公司,北京)用于采集MC-C^4^D装置的输出信号。旋涡混合器VORTEX-6(其林贝尔仪器制造有限公司,浙江)用于样品配制,智能手机HUAWEI P30Pro(华为,广东)用于拍照记录自由流电泳分离室中蛋白质条带分布情况。超纯水系统(SG Water公司,德国)用来生产电导率低至0.055 μS/cm的去离子水。

### 1.2 试剂

藻蓝蛋白(C-PC, *M*_r_ 40 kDa, pI 5.2)购自上海生物化工股份有限公司。牛血红蛋白(Hb, *M*_r_ 64.5 kDa, pI 6.8)购自中国医药集团有限公司。载体两性电解质(pH 3~10,固体含量40%)购自上海伯楷安生物科技有限公司。羟丙基甲基纤维素(hypromellose, HPMC)购自北京百灵威科技有限公司。氢氧化钠、磷酸、KCl、甘油等化学试剂均为分析纯,购自中国医药集团有限公司。

### 1.3 溶液的制备

称取一定量的KCl并在干燥器中干燥2 h。之后,在天平上称量7.456 g KCl,并将其溶解在去离子水中,配制50 mmol/L的KCl原液2 L。用去离子水稀释原液,得到0.05~20 mmol/L(相当于0.0075~2.776 mS/cm,温度为25 ℃时)的一系列KCl标准溶液。放入试剂瓶中备用,用于MC-C^4^D装置的标定和性能测试。

在研究FFE等电聚焦过程实验时,载体缓冲溶液组成为10%(v/v)甘油、3%(v/v)载体两性电解质、0.3 g/L HPMC,其余成分为去离子水;配制120 mL上述载体缓冲溶液,将15 mg藻蓝蛋白和15 mg牛血红蛋白溶于载体缓冲溶液,放入试剂瓶中备用。

取0.614 mL的磷酸(分析纯),加去离子水定容,配制100 mL的100 mmol/L的磷酸溶液作为阳极液;取0.4 g的氢氧化钠(分析纯),加去离子水定容,配制100 mL的100 mmol/L的氢氧化钠溶液作为阴极液。

### 1.4 MC-C^4^D装置

图1是MC-C^4^D装置非接触电导检测模块示意图,包括非接触电导检测池阵列、交流激励源、激励信号模拟开关、接收信号模拟开关、信号处理模块、数据采集卡和计算机,其中非接触电导检测池阵列包含16个电导检测池,每个电导检测池又由激励电极(a)、接收电极(b)、绝缘测试管道(c)和屏蔽单元(d)组成。在计算机上开发的自动测量软件控制下,交流激励源经激励信号模拟开关分时加载到各检测池的激励电极上,而在各接收电极上拾取的接收信号同样在自动测量软件的控制下经接收信号模拟开关同步分时连接到信号处理模块,信号处理模块对接收信号进行电流/电压转换、放大以及二极管峰值整流处理之后输出直流电压信号至数据采集卡,最后将经数据采集卡数字化的信号输入计算机由自动测量软件进行处理。由于交流激励源和接收信号都为双极性信号以及为了减小模拟开关接入对C^4^D检测性能的影响,这里采用了可双极性供电和具有低导通电阻的模拟开关。另外,每个电导检测池都设有屏蔽单元,起到减小电导检测池的极间干扰和外部干扰的目的。

**图1:**
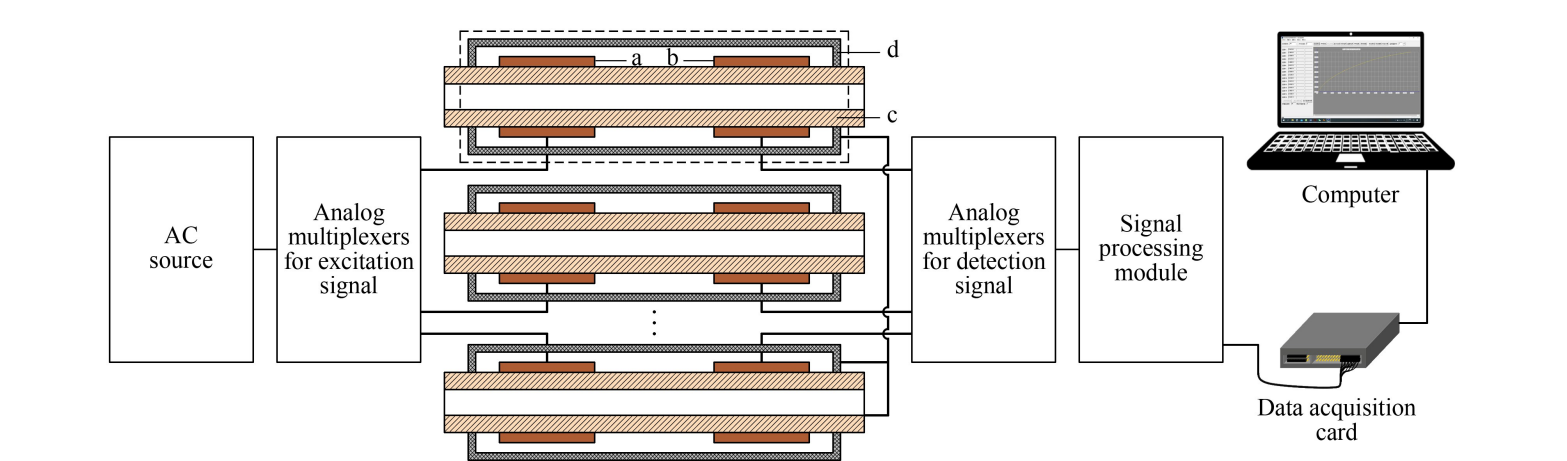
MC-C^4^D装置非接触电导检测模块示意图

考虑到FFE装置通道数多为16的倍数,单个电导检测模块设置为16个电导检测池(即16通道),当FFE装置通道数大于16通道时,则根据总通道数目并行设置多个电导检测模块。这种设计方式不但大大减小了MC-C^4^D装置的体积,而且简化了装置的电路系统,同时使得MC-C^4^D装置易于安装调试并减小了制作成本。

自动测量软件如图2所示,软件采用Visual Studio平台开发,适用于1~96通道MC-C^4^D装置,单通道最高采样频率最高可达20 Hz,具有连续测试、FFE测试和标定测试以及测试结果查询与显示等功能。由于C^4^D检测器直接输出结果是非线性的,因此自动测量软件在标定测试结束后对每个通道的测量结果都进行了6次多项式拟合修正,从而实现了C^4^D检测器的线性输出。

**图2:**
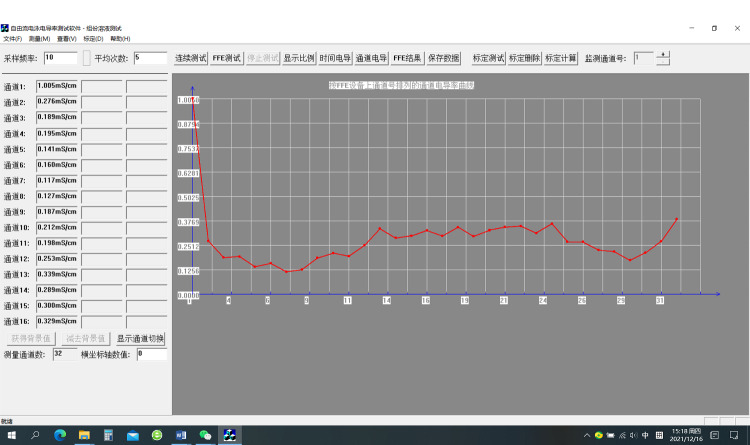
自动测量软件界面图

图3是32通道MC-C^4^D装置示意图,它由2个电导检测模块、箱体、数据采集卡和计算机组成,其中检测模块设置在箱体上半部分内,数据采集卡则放置在箱体的下半部分内并经数据线与计算机连接。MC-C^4^D装置的核心是电导检测模块,它由上盒盖(a)、电路板(b)、检测池阵列模块(c)和下盒盖(d)组成,其中检测池阵列模块(c)又由盖板(c_1_)、检测池阵列(c_2_)和阵列式通道槽板(c_3_)组成,单个电导检测模块尺寸为23 cm×8.4 cm×3.5 cm。检测池阵列(c_2_)是电导检测传感单元,它由16个电导检测池构成,每个电导检测池又由外壳、PEEK管、激励电极和接收电极组成。这里,外壳长度6.5 cm; 聚醚醚酮(PEEK)管内径0.1 cm,外径0.16 cm,长度11 cm;激励和接收电极长度均为2.5 cm;激励和接收电极间距为0.8 cm。制作时,根据上述参数在PEEK管上将铜箔胶带手动紧密绕制2圈,用这种方法分别绕制激励电极和接收电极,装进外壳并用胶水固定。上盒盖(a)、下盒盖(d)、盖板(c_1_)和阵列式通道槽板(c_3_)均采用金属材料制作,安装好后与电路板的地线连接,起到了屏蔽电导检测池的极间以及外部干扰作用,提高了MC-C^4^D装置的抗干扰能力。MC-C^4^D装置中激励信号的频率是110 kHz,采用5 V电源供电。

**图3:**
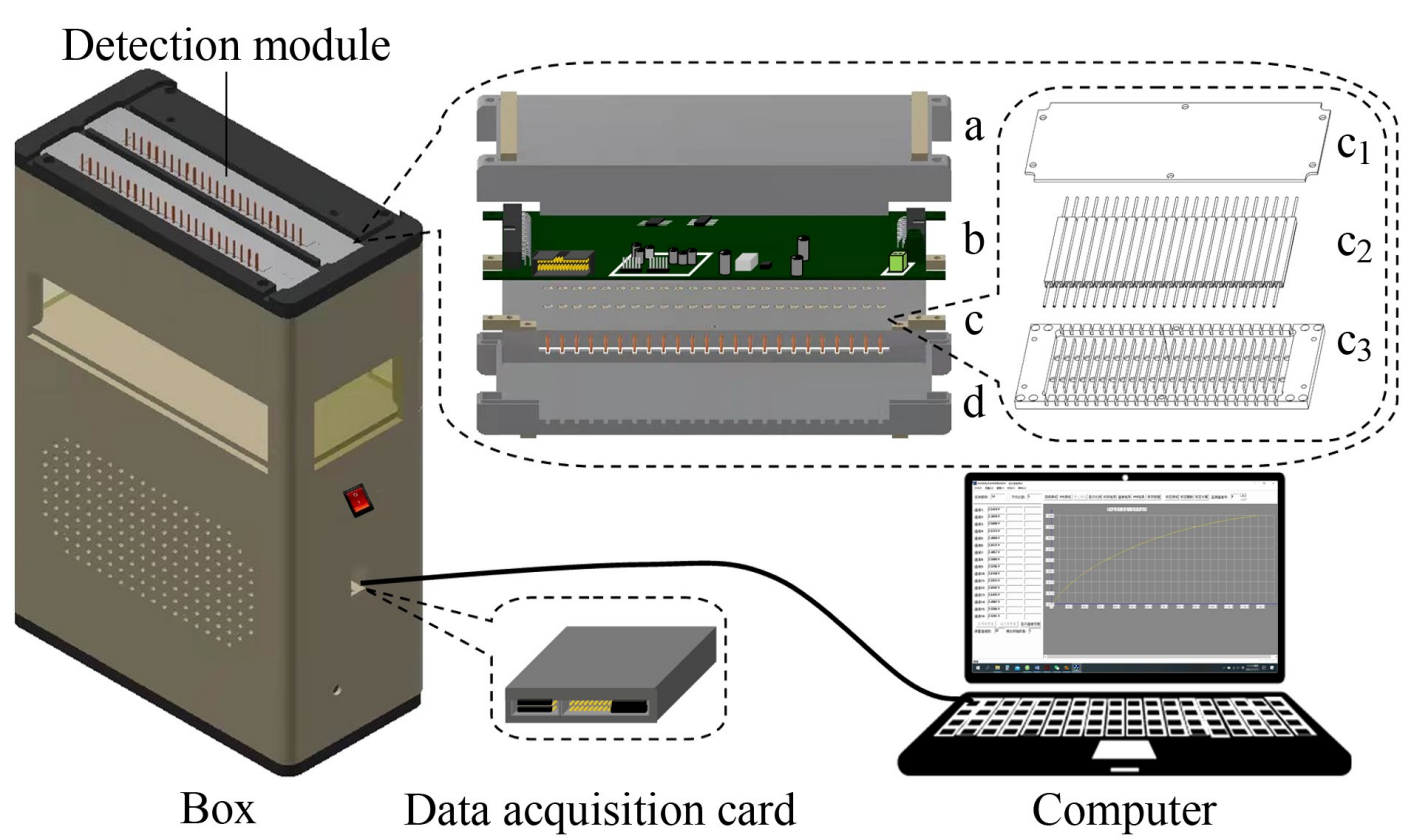
32通道MC-C^4^D装置示意图

### 1.5 标定测试和FFE实验操作方法

将MC-C^4^D装置串联接入FFE装置管路系统中,如图4所示。在开始测试前用去离子水将FFE装置管路冲洗多次,使得MC-C^4^D装置检测到的各个通道溶液电导率均较低(一般小于0.005 mS/cm)时,表明清洗完成。

**图4:**
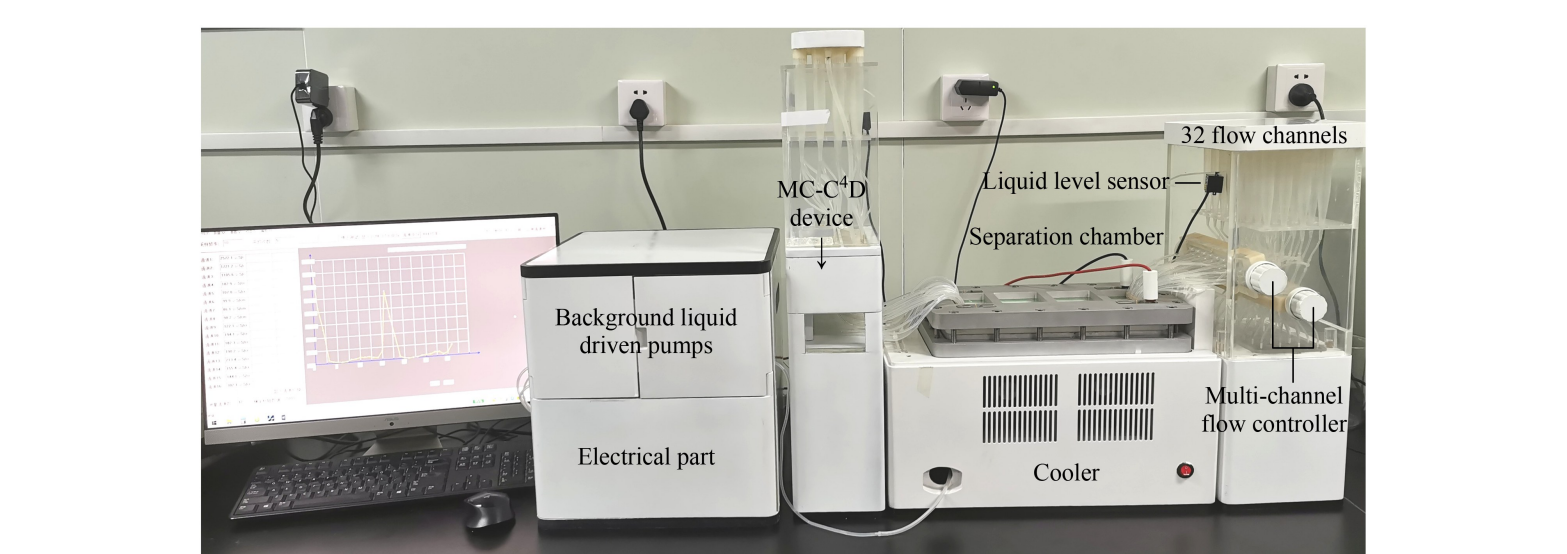
MC-C^4^D装置应用于RFFIEF仪器

标定测试 调节多通道流量控制器,打开分离室入口,关闭废液出口,将120 mL的0.1 mmol/L的KCl标准溶液从靠近离子交换膜一侧的重力平衡管中缓慢加入,在另一侧的重力平衡管用注射器抽出空气,直到32个重力平衡管中的液面高度一致,表明分离室和MC-C^4^D装置中已经没有气泡。在FFE装置控制软件界面打开背景液驱动泵,设定载体缓冲液在分离室往复流动的速度约为1 mL/min每个进液口,分离室温度为25 ℃,温度稳定后使用MC-C^4^D自动测量软件(见图2)对各个通道进行连续测试,待输出电压值稳定后切换到标定测试,获得各通道在这种标准溶液下的电导率检测结果。结束后将装置内的溶液排出,用去离子水将装置冲洗干净并排空。接着按照浓度从低到高依次更换标准溶液重复上述操作,获得各通道在不同浓度标准溶液下的电导率检测结果。测试完成后点击标定计算,使用多项式拟合法将各个测试点拟合出平滑曲线并获得修正系数,结果保存在计算机中。

FFE实验操作 调节多通道流量控制器,打开分离室入口,关闭废液出口,将120 mL溶有15 mg藻蓝蛋白和15 mg牛血红蛋白的载体缓冲液从靠近离子交换膜一侧的重力平衡管中缓慢加入,溶液沿着离子交换膜流入分离室,在另一侧的重力平衡管用注射器抽出空气,直到32个重力平衡管中的液面高度一致。在FFE装置控制软件界面打开背景液驱动泵,设定载体缓冲液在分离室往复流动的速度约为1 mL/min每个进液口,分离室温度为10 ℃。将100 mmol/L磷酸溶液和100 mmol/L氢氧化钠溶液分别作为阳极和阴极的电极液,打开电极液驱动泵,电极液在电极室中循环,流速为5 mL/min。温度稳定后使用MC-C^4^D自动测量软件进行在线电导率检测,打开分离电场电源,首先设定在恒功率模式25 W运行60 min,然后采用恒电压模式1000 V运行30 min。等样品被充分等电聚焦分离后,待载体缓冲溶液和样品都流到靠近离子交换膜一侧的重力平衡管中时,关闭背景液驱动泵,调节多通道流量控制器,关闭分离室入口,打开废液出口,载体缓冲溶液和已经实现分离的各个样品组分在重力的作用下流入回收器,完成整个过程。实验之后要使用大量去离子水冲洗装置,防止污染。

## 2 结果与讨论

### 2.1 MC-C^4^D装置性能

取1.3节中配制的KCl标准溶液,在一天内重复测量3次,得到MC-C^4^D装置的检测范围、相对标准偏差(RSD, *n*=3)、检出限(limit of detection, LOD)、相对误差(relative error, RE)和通道测量相对偏差。检测范围是0.015~2.5 mS/cm, RSD小于2.31%,测试结果表明装置检测范围较大,重复性好。随着KCl溶液逐渐稀释,对应溶液的电导率逐渐降低,当输出的结果和噪声幅度的比值为3倍时(*S/N*=3),记录此时KCl溶液对应的电导率,得到MC-C^4^D装置的LOD为0.002 mS/cm。跟标准溶液电导率相比较,MC-C^4^D装置的电导检测结果RE小于3.03%,另外通道间测量相对偏差小于1.60%,这些都说明所开发的装置通道间测量相对偏差较小,测量准确性高。

### 2.2 实验结果分析

图5是在RFFIEF运行期间分离室中蛋白质条带的照片。图5a是等电聚焦开始0 min时分离室的照片,此时在分离室中看不出任何差异。图5b是等电聚焦开始后30 min时分离室的照片,从图中可以发现在下方靠近阳离子交换膜的位置,有较弱的藻蓝蛋白条带。随着电泳时间延长,藻蓝蛋白和血红蛋白的条带越来越明显,如图5c为电泳开始后60 min时藻蓝蛋白和血红蛋白条带聚焦状况。当电泳时间达到90 min后,蛋白质条带变得更加明显,但是其所处位置和60 min时相比变化不大,这说明此位置pH值与其pI值相等,藻蓝蛋白和血红蛋白已经充分等电聚焦(见图5d)。

**图5:**
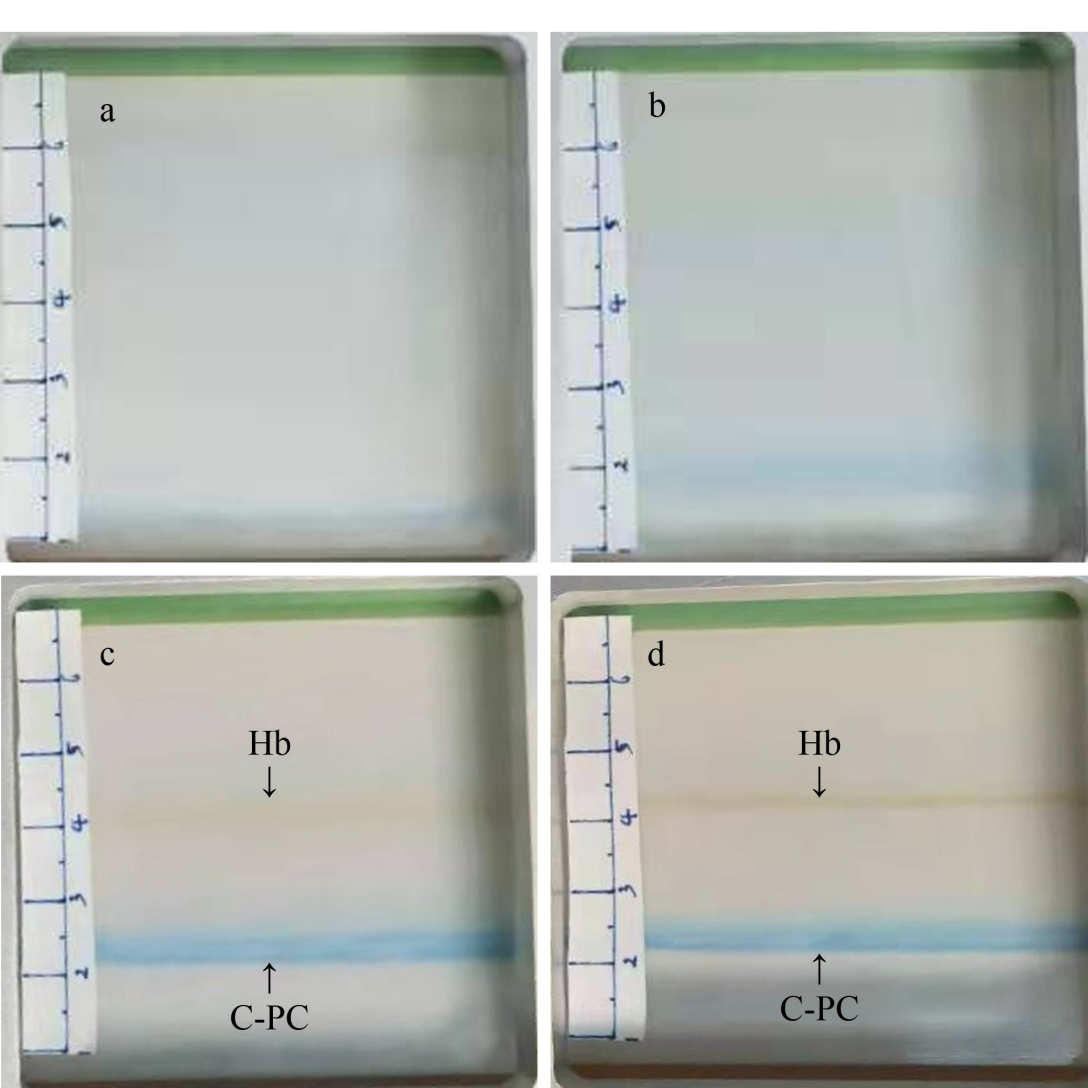
在RFFIEF运行期间分离室中蛋白质条带的照片

图6是10 ℃时MC-C^4^D装置在RFFIEF运行过程中对32个通道进行组分溶液电导率的自动检测结果。在等电聚焦开始0 min时,缓冲液和蛋白质均匀混合后第一次经过分离腔后,离子在电场中未达到平衡装填且pH梯度尚未完全形成,不同通道溶液的电导率差异较小,整体分布较为平缓。当实验进行在电泳30 min时,蛋白质逐渐在阳极端开始富集,蛋白质在向pI点聚集的过程中自身的净电荷量逐渐减小,这意味着其导电性逐渐减小,此时靠近阳极端通道溶液的电导率明显减小。在电泳聚焦60 min时,蛋白质富集明显,对应聚焦区域通道内溶液电导率进一步减小,同时靠近电极端离子存在堆积,导致相应通道内溶液电导率增大。此时最小电导率值出现在第7通道,这说明蛋白质主要富集在第7通道附近。电泳聚焦90 min时,对应聚焦区域内通道溶液电导率不再减小,但是聚焦区域有所减小且最小电导率值出现在第8通道附近,与60 min聚焦结果相比蛋白聚焦区域略有偏移,此时可以认为蛋白聚焦完成。

**图6:**
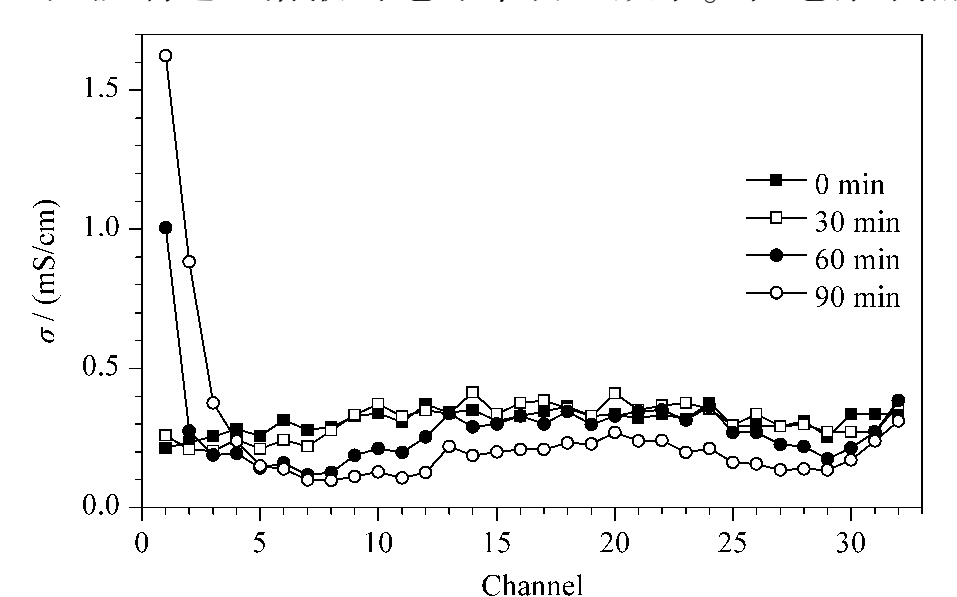
采用图4的MC-C^4^D装置对RFFIEF的32个通道中 载体缓冲溶液电导率进行自动检测的结果

### 2.3 与其他方法的比较

将本文开发的MC-C^4^D装置与商用接触式电导检测装置和单通道C^4^D装置进行比较,结果见表1。

**表1:** MC-C^4^D装置与DDS-307和单通道C^4^D装置性能的比较

Method	Multi-channel	Automation	Contactless	Detection range/(mS/cm)		LOD/(mS/cm)	RSD/%	Ref.
DDS-307	No	No	No	0	-100	-	<1.50	[26]
Single-channel C^4^D	No	No	Yes	0.01	-1000	-	-	[27]
MC-C^4^D	Yes	Yes	Yes	0.015	-2.5	0.002	<2.31	herein

从表1中可知,MC-C^4^D装置通道多,测量自动化程度高,因此其在多通道自动检测场景下拥有独特的优势。虽然MC-C^4^D装置测量范围不及DDS-307和文献^[27]^中的单通道C^4^D,但其已经可以满足FFE装置的要求。另外,我们知道,DDS-307是依赖手动切换挡位或者检测探头来达到0~100 mS/cm测量范围的,而单通道C^4^D要实现0.01~1000 mS/cm测量范围也同样需要手动更换检测头。

## 3 结论

本文发展了一种MC-C^4^D装置,开发了自动测量软件。MC-C^4^D装置采用了并行分时的非接触电导检测技术,实现了FFE装置对各组分溶液电导率的实时在线检测功能。为了验证MC-C^4^D装置的性能,采用配制的KCl标准溶液作为检测对象对其进行了标定和测试。实验结果表明,MC-C^4^D装置检测范围较大、LOD低、重复性好、准确性高以及通道间测量相对偏差小。最后还将MC-C^4^D装置成功应用于往复式RFFIEF在蛋白质聚焦过程中对各组分溶液电导率的实时在线检测。研究结果表明MC-C^4^D装置具有性能好、体积小、电路系统简单、安装调试容易和成本低廉等优点,可在多通道测量、在线检测和过程监测中发挥重要作用。
